# Translational potential of base-editing tools for gene therapy of monogenic diseases

**DOI:** 10.3389/fbioe.2022.942440

**Published:** 2022-08-10

**Authors:** Vasiliy V. Reshetnikov, Angelina V. Chirinskaite, Julia V. Sopova, Roman A. Ivanov, Elena I. Leonova

**Affiliations:** ^1^ Department of Biotechnology, Sirius University of Science and Technology, Sochi, Russia; ^2^ Department of Molecular Genetics, Institute of Cytology and Genetics, Novosibirsk, Russia; ^3^ Сenter of Transgenesis and Genome Editing, St. Petersburg State University, St. Petersburg, Russia; ^4^ Laboratory of Amyloid Biology, St. Petersburg State University, St. Petersburg, Russia; ^5^ Scientific Center for Genetics and Life Sciences, Sirius University of Science and Technology, Sochi, Russia

**Keywords:** base editing, dCas9, nCas9, prime editor, monogenic disease, gene therapy

## Abstract

Millions of people worldwide have rare genetic diseases that are caused by various mutations in DNA sequence. Classic treatments of rare genetic diseases are often ineffective, and therefore great hopes are placed on gene-editing methods. A DNA base–editing system based on nCas9 (Cas9 with a nickase activity) or dCas9 (a catalytically inactive DNA-targeting Cas9 enzyme) enables editing without double-strand breaks. These tools are constantly being improved, which increases their potential usefulness for therapies. In this review, we describe the main types of base-editing systems and their application to the treatment of monogenic diseases in experiments *in vitro* and *in vivo*. Additionally, to understand the therapeutic potential of these systems, the advantages and disadvantages of base-editing systems are examined.

## Therapy for genetic diseases

To date, ∼6,500 genetic diseases with annotated phenotypes have been identified and affect more than 100 million people worldwide (Nguengang Wakap et al., 2020). Treatments of such diseases have been limited mostly to symptomatic and supportive care. The main interventions into metabolic disorders are aimed at substrate restriction, replacement of deficient products, and inhibition of formation and removal of toxic metabolites (Yue et al., 2019). Enzyme replacement therapy, pharmacotherapy, and hematopoietic stem cell transplantation are used for these purposes (Chen and Altman, 2017; Li, 2018; Taylor et al., 2019). Genetic therapeutic strategies include gene replacement therapy, which requires targeted transfer of exogenous genetic material into human cells; mRNA correction (an antisense oligonucleotide, small interfering RNA, microRNA, or RNA editing); *cis*-regulation therapy; and gene-editing technology (Chen W. et al., 2020; Matharu and Ahituv, 2020; Reshetnikov et al., 2022). Recent advances in gene therapy are based on the use of nucleases such as ZFN, TALENS, and Cas9, which can precisely introduce double-strand breaks, that are repaired by the cell’s repair systems (Gaj et al., 2013; Guo et al., Forthcoming 2021). Nevertheless, the numerous off-target effects associated with DSBs, delivery challenges, and immunogenicity preclude the use of these tools in clinical practice (Cui et al., 2021; Guo et al., Forthcoming 2021), despite it has been used in clinical trials (Ou et al., 2020; Frangoul et al., 2021).

Recent advances in gene-editing technology made it possible to edit DNA without a DSB. This approach became feasible after a catalytically inactive DNA-targeting Cas9 enzyme (dCas9) was obtained, which together with single guide RNAs allows to localize effector domains to specific DNA sequences to either repress (CRISPRi) or activate (CRISPRa) transcription of a target gene(s) (Gilbert et al., 2014). CRISPRa has been successfully employed to treat diseases and eliminate haploinsufficiency in mice (Matharu et al., 2019; Colasante et al., 2020). Aside from the inactive Cas9, nCas9 has been obtained, which has a nickase activity and can create only a single-strand break at target sites (Cong et al., 2013). Fusion of nCas9 and APOBEC1 cytidine deaminase or TadA adenine deaminase has helped to devise cytosine and adenine base-editing systems, respectively (Rees and Liu, 2018). These tools can edit approximately 60% of known pathogenic mutations (Rees and Liu, 2018). Until recently, this state of affairs has been a shortcoming of the editors in question, but the development of prime editing tools, which can correct various types of mutations (transversion, insertion, or deletion), has removed these limitations (Anzalone et al., 2019). Here we describe the results of *in vitro* and *in vivo* research on animal models of rare genetic diseases, the main prospects and shortcomings of these tools, and current progress in their clinical application.

### Evolution of DNA-editing systems

The CRISPR-Cas9 system is an adaptive-immune-system component in bacteria and archaea and targets viral or plasmid dsDNA molecules (Wiedenheft et al., 2012). In laboratory practice, the most widely used Cas9 nuclease is Cas9 from the bacterium *Streptococcus pyogenes* (*Sp*Cas9) (Sander and Joung, 2014); however, some other analogs, for example, *Staphylococcus aureus* Cas9 (*Sa*Cas9) can also be used (Cebrian-Serrano and Davies, 2017; Matharu et al., 2019). The Cas9 nuclease is directed by guide RNAs (either a complex of tracrRNA with crRNA or a fusion single guide RNA) to a target dsDNA sequence containing a short stretch of nucleotides (downstream of the target sequence) termed the protospacer adjacent motif or PAM (for SpCas9, the PAM is 5′-NGG-3′, where N stands for any nucleotide). Upon recognition of a PAM and binding to the target sequence, DNA opens and the “R-loop” is formed (Jore et al., 2011). Cas9 activates and using RuvC-like and HNH domains makes two nicks in two complementary strands at the target locus, resulting in a DSB (Jinek et al., 2012). The CRISPR/Cas9 system has opened up numerous opportunities for genome editing in different organisms, and now there are many reports on its various applications [for review see (Mengstie and Wondimu, 2021)]; in particular, this system is used to create animal models of human diseases (Leonova and Gainetdinov, 2020). It has found many applications in biotechnology, including cracking the challenge of antibiotic resistance (Matharu et al., 2019; Novick, 2021, 202; Zohra et al., 2021). For instance, the use of a CRISPR-Cas9 system targeted against resistance genes has helped to reduce the resistance to β-lactames in *E. coli* (Kim et al., 2016) and *K. pneumonia* (Hao et al., 2020) and to lower the number of antibiotic-resistant *E. faecalis* strains (Rodrigues et al., 2019).

The CRISPR-Cas12 system is another editing system of bacterial origin for targeted DSB introduction. Cas12 nucleases are guide RNA–targeted DNA-specific endonucleases recognizing a PAM (for Cas12 proteins, it is usually T-rich, for example, for Cas12a, the PAM is 5′- TTTV-3’, where V is for G/C/A) (Chen P. et al., 2020). Unlike Cas9, Cas12 needs only one short crRNA for targeting (Zetsche et al., 2015). By now, at least 11 types of Cas12 proteins have been discovered: Cas12a (formerly known as Cpf1), Cas12b, Cas12c, Cas12d, Cas12e, Cas12f (also known as Cas14), Cas12g, Cas12h, Cas12i, Cas12j, and Cas12k (Tong et al., 2021). It has been shown that upon recognizing a target and making the first cut, Cas12 proteins stay and exert a nonspecific endonuclease activity toward surrounding DNA molecules, which is called collateral activity (Chen et al., 2018). Cas12 proteins have been widely used for gene editing and transcriptional regulation [for review see (Tong et al., 2021)]. In addition, with Cas13, the Cas12 nuclease is employed in (mostly viral) nucleic-acid detection systems like DETECTR or SHERLOCK (Gootenberg et al., 2018); in particular, these systems have been proposed for COVID-19 detection (Safari et al., 2021).

It should be noted that Cas9 introduces DSB with the formation of blunt ends, while Cas12 introduces sticky ends. Anyway, both systems can activate similar repair systems: Ku-dependent non-homologous DNA end joining (NHEJ), a Polymerase θ-mediated end joining (TMEJ or microhomology-mediated end joining (MMEJ)), and homology-directed repair (HDR). The molecular mechanisms of preference in cellular repair post-CRISPR/Cas9 cleavage are still unclear (Wyatt et al., 2016). The choice what kind of repair pathway will be activated depends on many factors, such as the phase of the cell cycle, chromatin structure and the CRISPR/Cas construction (Leonova and Gainetdinov, 2020; Vítor et al., 2020). For example, MMEJ seems to be most active during the M and early S phases in dividing cells (Yanik et al., 2018). The most important factor in determining which double-strand break repair pathway will be used is whether or not the 5’ termini of broken ends are resected. Ends with little (about 10 n.t.) or no single stranded overhang are typically rejoined by Ku-dependent NHEJ. In contrast, TMEJ assumes prominence as the extent of 5′ > 3′ resection exceeds 45 nt (Yousefzadeh et al., 2014; Yanik et al., 2018). Repair of DSB by different mechanisms leads to many random indels thereby making the DNA-editing process inaccurate. To address the challenge of making single-nucleotide DNA editing precise and efficient, Cas12 and Cas9 proteins have been modified (nuclease domains activities have been either fully eliminated or turned to nickases) and then fused with adenosine or cytidine deamination enzymes.

First *Sp*Cas9 nickases (endonuclease variants where Cas9 cuts either the paired (targeted) or unpaired (non-targeted) DNA target strand but not both) have been obtained by introducing amino acid (a.a.) substitutions into *Sp*Cas9 nuclease domains: residue D10 in the RuvC-like domain or residue H840 in the HNH domain has been replaced with alanine (Sapranauskas et al., 2011). Incubation of these variants of the *Sp*Cas9 nuclease (hereafter referred to as nCas9) in complex with guide RNA and plasmid DNA results in nicked open circular plasmids, whereas wild-type *Sp*Cas9 produces a linear DNA product. Furthermore, it has been found that the RuvC-like domain cleaves an unpaired DNA strand, while HNH cleaves the paired strand (Jinek et al., 2012). When both mutations (D10A and H840A) are introduced into *Sp*Cas9, the nuclease activity is eliminated, but the targeting activity remains. This catalytically inactive nuclease is called dead Cas9 or dCas9.

### Cytidine base editors

The cytidine deaminase reaction in DNA leads to the cytidine-to-uridine transition giving rise to a functional G-to-A substitution. AID/APOBEC cytidine deaminases are well known and are normally found in jawed vertebrates. These enzymes can bind and deaminate RNA and single-strand DNA (ssDNA). In humans, this family includes several cytidine deaminases: AID, APOBEC1, APOBEC3 (a subfamily with seven members: A, B, C, D, F, G, and H), APOBEC2, and APOBEC4 (Salter et al., 2016). Apart from APOBEC, in genetic engineering, researchers use activation-induced deaminase (AID) (from vertebrates) and lamprey CDA1 and CDA1-like proteins (Muramatsu et al., 1999; Pancer et al., 2004). Despite the low sequence identity between human AID/APOBEC and CDA1 (and CDA1-like) proteins and because all these enzymes are functional cytidine deaminases involved in adaptive immunity, it is believed that CDA1 and CDA1-like proteins may be affiliated with the AID/APOBEC family of proteins (Holland et al., 2018).

**FIGURE 1:**
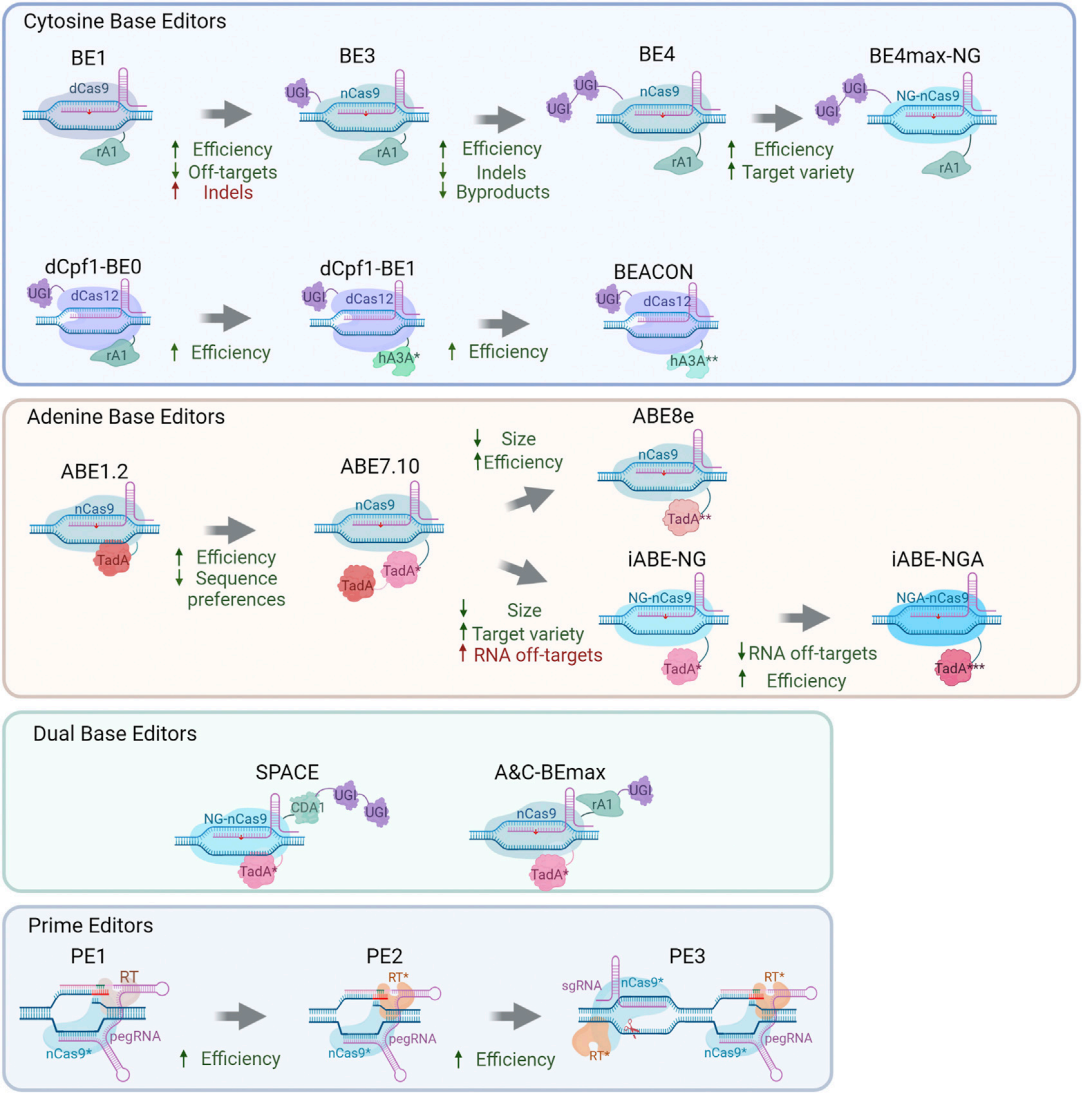
| A brief overview of base-editing systems.

The first Cas9-targeted DNA-specific cytidine base editor (CBE) was created by Komor and others in 2016 (Komor et al., 2016). Rat APOBEC1 (rAPOBEC1) was fused to the N terminus of dCas9 through the XTEN linker resulting in rAPOBEC1-XTEN-dCas9 chimeric protein (Figure 1). This editor manifested more than 50% effectiveness of DNA deamination *in vitro*; however, *in vivo*, its effectiveness is drastically lower (0.8–7.7%) due to the cellular response to U-G heteroduplex DNA: activation of uracil DNA glycosylase (UDG), which catalyzes the removal of U from DNA in cells and initiates base excision repair (Kunz et al., 2009), thus leading to С recovery at the target site.

To address this problem, a UDG protein inhibitor (UGI) from bacteriophage PBS1 (Mol et al., 1995) has been fused to the C terminus of BE1 and utilized as a second-generation base editor (BE2) (Komor et al., 2016). Deamination efficiency of BE2 (rAPOBEC1-dCas9-UGI) has been assessed *in vivo* on six genomic loci. The results were promising: a threefold rise (in comparison to BE1) led to ≤20% C-to-U conversion effectiveness in HEK293 cells.

Mismatch repair (MMR) machinery uses nicked heteroduplex DNA as a good substrate for PCNA loading and subsequent endonuclease activation on the incised strand (Pluciennik et al., 2010); therefore, introducing a nick into the nonedited DNA strand near the nucleotide mismatch (base-editing result) may increase the repair of the wild-type strand and elevate the amounts of edited DNA. Thus, to further increase the base editor conversion degree *in vivo*, the Cas9 nuclease should create a nick in the nonedited strand of target DNA. Base-editing efficiency of BE3 in human cells has turned out to be even higher than that of BE2 and in some cases reached 75% (Komor et al., 2016); however, a slightly increased indel rate was observed after BE3 treatment in comparison with BE1 or BE2. Off-target activity of this system was reported to be low and mostly due to Cas9 off-target effects.

In 2016, the Target-AID cytidine base editor was constructed by Nishida and others (Nishida et al., 2016). The first Target-AID system was based on the targeting activity of dCas9 and the cytidine deamination activity of an AID/APOBEC family protein: an AID lamprey ortholog called CDA1. Two proteins were fused through a long (100 a.a.) peptide linker. This system has shown only a 2% mutation rate in yeast cells (Nishida et al., 2016). To raise editing rates, Target-AID has gone through evolution similar to BE systems: firstly, dCas9 was replaced with nCas9(D10A) increasing effectiveness up to 35%. Next, this protein was fused to UGI, which raised the mutagenesis rate up to 74%. The latest Target-AID system acts in a similar fashion but not identically to BE3: in Target-AID, modifications were preferably introduced 15–19 bases upstream of the PAM (overlapping with the BE3 effective editing window); in contrast to rAPOBEC1, CDA1 seems to lack sequence preferences, and therefore the Target-AID system has good potential for therapeutic use owing to a wider range of target sequences.

PAM recognition by Cas9 is a factor lowering the practical potential of Cas-based systems by narrowing the spectrum of targets. To solve this problem, Kleinstiver and coworkers (Kleinstiver et al., 2015) have mutagenized Cas9 in the PAM recognition domain. The resulting mutants were named *Sp*CasVQR (containing D1135V/R1335Q/T1337R mutations) and *Sp*Cas9EQR (containing D1135E/R1335Q/T1337R mutations) and recognized respectively NGAN (also NGNG but with generally lower efficiency) and NGAG PAMs. Additionally, a quadruple mutant of *Sp*Cas9VRER (D1135V/G1218R/R1335E/T1337R) was obtained. It manifested the highest activity toward an NGCG PAM and minimal activity toward an NGG PAM (Kleinstiver et al., 2015). In 2018, Nishimasu with colleagues modified *Sp*Cas9 for nonclassic PAM recognition. Introduction of several mutations (R1335V/L1111R/D1135V/G1218R/E1219F/A1322R/T1337R) into *Sp*Cas9 (the obtained Cas9 variant was designated as *Sp*Cas9-NG) has led to relaxed 5′-NG-3′ PAM recognition (Nishimasu et al., 2018).

Kim with colleagues (Kim et al., 2017) have developed some BE3-modifications regarding PAM recognition. The *Sa*Cas9 nuclease is smaller than SpCas9 and recognizes another PAM: NNGRRT (Ran et al., 2015), thereby potentially expanding the number of available target sites for cytidine base editing. A nickase version of *Sa*Cas9 (*Sa*Cas9n) was fused with rAPOBEC1 and UGI, and this protein was named *Sa*BE3. The efficiency of this system on target sites in general exceeded that of BE3 (Kim et al., 2017). The *Sp*Cas9 protein of BE3 was replaced with above-mentioned mutated Cas9 proteins (VQR, EQR, or VRER Cas9 variants) to set up VQR-BE3, EQR-BE3, and VRER-BE3 systems, which should target NGAN, NGAG, or NGCG PAMs, respectively. The efficiency of editing by these proteins in HEK293 cells is up to 50% while having a low off-target activity (Kim et al., 2017). Mutating an APOBEC1 active-center residue (W80Y/F) narrowed the editing window to three nucleotides. Likewise, mutations in the substrate-binding domain of APOBEC1 (R126E or R132E) narrowed the editing window. Proteins with double mutations (W80Y R126E, W80Y R132E, or R126E R132E) have the editing window ∼2 nt wide, thereby showing more predictable and precise editing, whereas triple mutants have almost threefold lower maximal editing yields, with the editing window narrowed to almost two to one nucleotide (depending on the target locus). When combined, the two innovations (window-modulating mutations in APOBEC1 and VQR-BE3) allowed editing with a narrowed activity window and greater positional selectivity of target sites containing an NGA PAM (Kim et al., 2017).

In 2017, Komor and others (Komor et al., 2017) developed BE3s involving different AID/APOBEC family members (AID, CDA1, or APOBEC3G) to address the problem of sequence context preferences of rAPOBEC1. It was reported that AID-BE3 and CDA-BE3 are efficient when the nucleotide one bp upstream of the target C is G; however, overall (non-GC) editing rates were lower in comparison with BE3. Furthermore, deamination by AID-BE3 and CDA-BE3 was more accurate (the product was purer) in comparison with BE (Komor et al., 2017). Not only deaminases but also the mutual position of BE parts could influence efficiency, accuracy, and robustness of cytidine deamination. For instance, extending the linker length to 32 a.a. Between proteins nCas9 and rAPOBEC1 gave a 1.2-fold increase in reaction efficiency. Extending the linker length between nCas9 and UGI to 9 a.a. Led to a 1.3-fold decrease in non-T product formation, with no apparent changes in C-to-T editing. Insertion of another copy of UGI into the C terminus of BE3 induced a more than twofold increase in product purity relative to BE3. Combining these three improvements has led to the development of the fourth generation of base editors: BE4 (Komor et al., 2017). Compared to BE3, BE4 offers a 2.3-fold decrease in byproduct amounts as well as 2.3-fold lower indel formation.

Next, Rees with colleagues (Rees et al., 2017) modified BE3 to reduce off-target effects and created HF-BE3, a base editor containing high-fidelity Cas9 variant HF-Cas9 (containing four point mutations [N497A, R661A, Q695A, and Q926A] for elimination of nonspecific Cas9–DNA interactions). In comparison with BE3, HF-BE3 shows 37-fold less off-target editing with only a slight reduction in on-target editing efficiency. Successful delivery of the HF-BE3 system using ribonucleoproteins into the mouse ear and zebrafish embryo and generation of C-to-T substitutions *in vivo* has been reported (Rees et al., 2017).

In 2021, Liu and others (Liu et al., 2021) designed a cytidine editing system based on the *Neisseria meningitides* Cas9 (Nme2-Cas9) specific to cytidine dinucleotide PAM (N4CC), thus enlarging the target sequence pool and offering compact size (1,082 a.a.) and natural high fidelity. A cytidine base editor with *Nme*2-Cas9 was created by replacing n*Sp*Cas9 from the BEmax editor with a nickase version of *Nme*2-Cas9 (D16A). The obtained editor was designated as n*Nme*2CBE. Compared to n*Sp*-CBE, the newly developed editor showed comparable editing efficiency and a smaller amount of off-target products (Liu et al., 2021).

The problem of the size of base editors has been addressed differently: some researches propose to use orthologs of SpCas9 (Ran et al., 2015; Liu et al., 2021), but in ref. (Levy et al., 2020), another approach is utilized. Adeno-associated virus (AAV) delivery is size-limited; accordingly, for ABE or CBE systems to be split, it was decided to use a *trans*-splicing intein enabling CBE and ABE division into halves thereby enabling dual AAV packaging of base editors. The assembly of this split-intein CBE was conducted in several steps: fusion of each split DnaE intein half from *Nostoc punctiforme* (*Npu*) to each half of the original BE3, followed by dividing it within the SpCas9 sequence immediately before Cys574. This split base editor construct is called *Npu*-BE3 and has a good on-target base editing rate of approximately 34% in HEK293T cells. A BE4max-based *Npu*-BE4max construct has also been developed. Codon usage optimization and a nuclear localization signal (NLS) resulted in higher base-editing efficiency (44%) than that of *Npu*-BE4 involving IDT (Integrated DNA Technologies) codon optimization (22%). A rational version of the base-editing system for AAV delivery consisted of a spliced NLS- and codon-optimized APOBEC fused to the Cas9 nickase and UGI and is referred to as CBE3.9max (Levy et al., 2020). This base editor has gone through a number of modifications until optimized v5 AAV split-CBE3.9max manifested 56% base-editing efficiency in HEK293 cells. *In vivo* (in a mouse), this construct had organ-dependent moderate efficiency varying from 4% in skeletal muscles to 21% in the liver (Levy et al., 2020).

Besides Cas9, another Cas nuclease family member has been repurposed for targeted base editing, which is Cas12. Li and others have fused catalytically dead Cas12a from *L. bacterium* (dLbCas12) with rAPOBEC1 and a uracil DNA glycosylase inhibitor; thus, a dCas12 targeted base editor was obtained (Li et al., 2018). It showed high editing activity (up to 70% efficiency toward some cytosines). In mammalian cells, efficiency dropped down to 20% on average. The main editing window of this base editor ranges from position 8 to 13 (assuming that the base next to the PAM is position 1). Introducing mutations W90Y and R126E in APOBEC has narrowed the editing window to 10–12 positions of the spacer (Li et al., 2018). dCas12BE has undergone numerous modifications (Wang X. et al., 2020) starting from fusion to various AID/APOBEC family proteins: rAPOBEC1, hAPOBEC3A, hAPOBEC3B, or hAID (referred to rA1, hA3A, hA3B, and hAID, respectively). hA3A-dCas12a-BE has the highest editing efficiency among the aforementioned nucleases. Later, mutations W98Y, W104A, and P134Y have been introduced into hA3A-dCas12a-BE, and relative efficiency has been assessed: hA3A^W104A^-dCas12a-BE, hA3A^W98Y/W104A^-dCas12a-BE, and hA3A^W104A/P134Y^-dCas12a-BE perform active editing. Next, similarly to ref. (Koblan et al., 2018), codons in dCas12-BEs have been optimized for mammalian expression. Editing windows of the obtained mutated hA3A-dCas12a-BE-ops were shown to be ∼15 bp long. Introducing the Y132D or Y130F mutation into the hA3A region of dCas12BE leads to editing-window narrowing, increased accuracy, and a lower frequency of indel formation. Consequently, hA3A_W104A/Y132D_-dCas12a-BE-op and hA3A^W98Y/W104A/Y130F^-dCas12a-BE-op were called BEACON1 and BEACON2, respectively, and have shown editing productivity similar to that of AncBE4max (while creating much fewer indels) in the cell. Furthermore, the BEACONs have been tested *in vivo*: C-to-T editing efficiency in mouse organs ranges from 51% to 71% (Wang X. et al., 2020).

In general, CBE architectures have gradually evolved to improve editing efficiency and product purity, to lower the indel rate, and to broaden PAM recognition specificity in a native environment of a target sequence.

### Adenine base editors

Another class of base editors is adenine base editors or ABEs. There are no natural adenine deaminases acting on DNA, and to make DNA adenine deamination possible, RNA-specific deaminases should be modified. Gaudelli with colleagues have devised an adenine base editor converting adenine to inosine in DNA, resulting in a T-to-G substitution (Gaudelli et al., 2017). They used directed evolution to create a DNA-specific form of RNA-specific adenine deaminase TadA. TadA is a tRNA adenine deaminase converting adenine to inosine (I) in the ssRNA of the anticodon loop of tRNA_Arg_ (Kim et al., 2006). Some APOBECs share homology with TadA, and it is reported that APOBECs possess RNA- and DNA-binding properties. Therefore, it has been hypothesized that some mutant TadA (TadA*) enzymes are able to bind and edit DNA. As a consequence, mutations A106V and D108N have been incorporated into the TadA deaminase, and the obtained protein has been subsequently fused through the XTEN linker to nCas9(D10A) and a C-terminal NLS (Figure 1). The resulting protein serves as the ABE1.2 DNA base editor. Editing efficiency in cells is only 3.2%, and editing is performed mostly at the fifth protospacer position (generally ∼fourth to ninth position, assuming that the PAM is positions 21–23) (Gaudelli et al., 2017).

This inefficient but working DNA-specific adenine editor has given rise to the evolution of ABE systems. Incorporation of mutations D147Y and E155V into TadA* (giving the ABE2.1 system via replacement of the precise version of deaminase) has led to a twofold to sevenfold increase in editing efficiency as compared with ABE1.2 at six genomic loci tested. An ABE2.6 variant with a prolonged XTEN linker (to 32 a.a.) has slightly higher (relative to ABE2.1) editing efficiency: 14%. Because normally, TadA operates as a homodimer (Losey et al., 2006), TadA* (version 2.1) has been fused to the N terminus of ABE2.1, and the efficiency of the obtained ABE2.9 system is 7.5% higher, resulting in an editing efficiency of 20%. Three new TadA mutations (L84F, H123Y, and I157F) have been applied to ABE2.9 to generate the ABE3.1 system showing 1.6-fold better performance than ABE2.9 does; however, a distinct sequence preference was observed. To solve this problem, various mutations were introduced into the TadA protein. Four mutations (H36L, R51L, S146C, and K157N) in ABE3.1 led to ABE5.1, which shows decreased editing efficiency in HEK293T cells. This intermediate system was modified by fusing wild-type TadA to the N terminus of ABE 5.1, thus giving rise to ABE 5.3 (with average editing efficiency of 39%) and broadening sequence compatibility. Introducing P48S into TadA* (5.3) resulted in the ABE6.3 system with elevated average DNA-editing efficiency, by 1.3-fold. Mutations W23R, P48A, and R152P in ABE6.3 resulted in the ABE7.10 system showing improved editing efficiency, up to 58%, at six loci in HEK293 cell lines; this performance is 29-fold better than that of the ABE1.2 system. Subsequent ABE7.10 analysis has revealed that the indel percentage is extremely low (<0.1%) and off-target activity is almost absent, suggesting that systems eliminating inosine from DNA are less active than these toward uracil. These seven sequential evolution rounds of ABEs are giving the scientists a lot of information about the editing principles of ABE systems (Gaudelli et al., 2017).

Another approach to addressing the editing efficiency problem is used in ref. (Koblan et al., 2018). These researchers found that the stability and magnitude of base editor expression are factors influencing base modification. Codon usage optimization and NLS presence were hypothesized as factors impairing base modification. It was demonstrated that bipartite NLS (bpNLS) presence at both the N and C termini of BE4 induces a 1.3-fold improvement in its editing efficiency. Bis-bpNLS BE4 with GenScript codon usage was named BE4max and had 1.8-fold higher editing efficiency as compared to bis-bpNLS BE4 with IDT codons, and manifested approximately 89% editing efficiency in HEK293 cells. An analogous approach has been chosen for adenine base editor ABE7.10: SV40 NLS substitution by bis-bpNLS improved editing efficiency 1.5–2.0-fold, and GenScript codon optimization yielded 1.3- to 7.9-fold higher editing efficiency of this base editor called ABEmax in comparison to IDT. ABEmax has remained an extremely accurate and robust editor, but its indel rate is 1.7% compared to <0.1% of ABE7.10 (Koblan et al., 2018).

Reports of the development of SpCas9-NG along with ABEmax have inspired Huang and others (Huang S. et al., 2019) to develop a fusion ABEmax-SpCas9-NG system called NG-ABEmax. High editing efficiency and NG-PAM recognition offer great potential for splice site modification in order to modulate RNA splicing in the cell. It was demonstrated that ABEmax-NG effectively recognizes all types of NG(N) PAMs and efficiently performs DNA editing *in vitro* and *in vivo*, whereas ABEmax recognizes the classic NGG PAM with high efficiency and the NGA PAM with modest efficiency (Huang S. et al., 2019). Meanwhile, T. Huang with colleagues have created analogous systems based on other Cas9 nucleases with altered PAM recognition: VRQR-SpCas9 (PAM: NGA) and VRER-SpCas9 (PAM: NGCG) (Kleinstiver et al., 2015, 2016), yielding VRQR-ABEmax and VRER-ABEmax, respectively (Huang T. P. et al., 2019). These editors were tested in HEK293 cells at six genomic loci. VRQR-ABEmax manifested 35% editing efficiency, being 3.2-fold better than ABEmax. VRER-ABEmax showed conversion efficiency averaging 40%: a 7.0-fold improvement over ABEmax (Huang T. P. et al., 2019). ABE7.10 evolution continues, and in 2020, two papers got published describing another generation of ABEs. Using phage-assisted noncontinuous and continuous evolution (Richter et al., 2020), investigators have obtained a next-generation ABE: ABE8e, which contains eight additional mutations leading to a dramatic activity boost as compared with ABE7.10 without increasing off-target activity.

Gaudelli and others (Gaudelli et al., 2020) have evolved ABE7.10 into 40 new ABE8 variants. Compared to ABE7.10, ABE8 performs ∼1.5-fold more efficient editing at canonical positions (A5–A7) in the protospacer and ∼3.2-fold more efficient editing at noncanonical positions (A3–A4 and A8–A10). Additionally, ABE8 recognizes classic PAM (NGG), and its editing efficiency is 4.2-fold higher at non-NGG PAM variants as compared to ABE7.10. ABE8s have base-editing capacity even at sites previously difficult to target. ABE8s can achieve 98–99% target modification in primary T cells, meaning that these editors are a promising tool for cell therapy applications (Gaudelli et al., 2020).

Some ABEs perform off-target RNA editing. It has been hypothesized that the reason lies in the wtTadA domain of the editor. In ref. (Grünewald et al., 2019), researchers deleted the wtTadA domain of ABEmax thereby obtaining the miniABEmax construct. The undesirable off-target RNA editing declined but not dramatically: 1.5-fold. Introducing mutation K20A/R21A or V82G into TadA* led to lowering of nontarget adenine modification rates with on-target efficiency rates being slightly higher for miniABEmax (V82G). There was also an interesting observation that miniABEmax (V82G) possesses an imprecise C-to-G base-editing activity within the editing windows of some DNA on-target sites (Grünewald et al., 2019).

Similarly to ref. (Grünewald et al., 2019), Xu and others (Xu et al., 2021) have developed a TadA-less adenine base editor using the SpCas9-NG nuclease. The heterodimeric adenine deaminase domain (ecTadA-ecTadA*) in ABE-NG either with the originally evolved ecTadA monomer or its high-fidelity version (ecTadA-V82G) for minimization the of the off-target RNA editing activity gave the miniABE-NG (iABE-NG) editor system. The on-target DNA editing activity of miniABE-NG is higher than that of ABE7.10-NG; however, miniABE (V82G)-NG has remarkably lower on-target editing activity when compared to ABE7.10-NG. An attempt to improve the on-target DNA editing efficiency of high-fidelity miniABE (V82G)-NG without increasing its low off-target RNA editing activity was made. The A56G mutation resulted in the miniABE (GG)-NG editor featuring completely restored on-target DNA editing activity with remaining low off-target activity (Xu et al., 2021).

### Dual base editors

Having both adenine and cytidine deamination activities in one system seems to be a nice and desirable prospect. One of the ways to do so is to combine an existing CBE and ABE. Such a dual deaminase has been devised by Grünewald and others (Grünewald et al., 2020). This system called SPACE (synchronous programmable adenine and cytosine editor) consists of the miniABEmax (V82G) editor fused with Target-AID CBE (Figure 1). SPACE can carry out A-to-G editing at 25 out of 26 genomic sites edited by miniABEmax-V82G alone, but cytidine editing is performed at all target loci as compared to Target-AID. The efficiency of adenine editing by SPACE is somewhat lower relative to miniABEmax-V82G (13% versus 18.1%, respectively), whereas C-to-T editing efficiency rates of SPACE and Target-AID are quite similar (22% versus 24%, respectively). The frequency of unwanted indels induced by SPACE is quite low (on average 1.44%) (Grünewald et al., 2020).

A similar approach was used in ref. (Zhang et al., 2020), i.e., a dual base editor system. The researchers fused ABE7.10 with BE3. Two deaminases were fused with nCas9 and UGI. For enhancing efficiency, a number of modifications were made: codon optimization was applied to hAID and TadA domains; two bipartite NLSes were added to the editor; a rigid 15-mer (EAAAKEAAAKEAAAK) linker was chosen for fusion; and finally, two copies of uracil DNA glycosylase inhibitor (UGI) were added thus resulting in the A&C-BEmax system. The A-to-G editing window of A&C-BEmax is not changed as compared to ABEmax, while the C-to-T editing window widened to 16 nucleotides in comparison with that of AID-B4 (10 nucleotides). A&C-BEmax is an efficient DNA editor showing simultaneously different A/C mutation rates on the same allele, varying from 2% to 30%, with the percentage of alleles bearing only C-to-T or A-to-G mutations varying from 5.3% to 82.6% and from 0.2% to 10%, respectively. Conversion rates are higher when adenines are at position 6 or 7. In HeLa cells at all examined targets, various base-editing efficiency (reaching 20–60% depending on target) is observed (Zhang et al., 2020).

### Prime base editors (PEs)

These are conceptually new base editors allowing to directly introduce any possible substitution (both transitions and transversions) into a desired site. The first PE (PE1) was created through a fusion of nSpCas9(H840A) with Moloney murine leukemia virus reverse transcriptase (RT) (Anzalone et al., 2019). nSpCas9(H840A) fused with RT is guided to target DNA using special prime editing guide RNA (pegRNA) (Figure 1). The latter has several functions: it guides the base editor to a target DNA, interacts with DNA, bears desirable base modifications, and primes the reverse-transcription reaction (by means of a primer-binding sequence or PBS). The principle underlying the base editing by PEs is the following: nSpCas9(H840A) guided to a target locus makes a nick, a 3′ ssDNA flap is bound by pegRNA PBS, and this strand serves as a primer for reverse transcription, which extends the 3′ ssDNA flap and incorporates pegRNA-coded base substitutions into the DNA strand. The 5′ flap is excised, and a 3′ flap-favored base is incorporated. Yeast application of this system shows only modest editing efficiency.

In the PE2 system, mutations D200N, L603W, T330P, T306K, and W313F are introduced into RT, resulting in thermostability and processivity improvement leading to a 1.6- to 5.1-fold higher mutagenesis rate relative to PE1 (Anzalone et al., 2019). To improve favorability of the repair of the nonedited strand, a strategy entailing nick introduction into the nontargeted strand has been proposed. The additional guide RNA has been suggested for directing the Cas9 H840A nickase (a part of the PE system) to incise the genomic DNA at a nearby site still not causing a DSB. Application of this method has manifested elevated editing efficiency, up to 55%, with nicks positioned at approximately 40–90 bp on the 3′ side of the pegRNA-induced nick (Anzalone et al., 2019).

Here we focus only on some modifications of the editing systems that have been utilized *in vitro* and *in vivo* to correct point mutations. A number of other BE, ABE, and PE modifications are listed in another review (Yang et al., 2021).

All in all, here we outlined the main events in the evolution of base editors from BE cytidine base editors (able to make one type of substitution in a strictly controlled sequence with off-target editing) to PEs able to edit multiple nucleotides at various loci with high efficiency and a low off-target rate.

### Site-directed DNA base editing for therapy of monogenic diseases

Correction of genetic point mutations via DNA-editing approaches has become widespread in animal models (*in vivo* research). Although these genome-editing tools have not yet been tested clinically, the data from animal research show their possible usefulness for the treatment of various rare monogenic diseases. The examples of *in vivo* and *in vitro*/*ex vivo* DNA editing for therapy of monogenic diseases are listed in Table 1, Table 2 respectively.

**TABLE 1:** *In vivo* DNA editing for therapy of monogenic diseases

Strain	Model	Delivery system	Editing systems	Target gene	Tissue	References
rd12 mice	Leber congenital amaurosis	AAV	NG-ABEmax	*Rpe65*	retina	Jo et al. (2021)
rd12 mice	Leber congenital amaurosis	Lentivirus	ABEmax	*Rpe65*	retina	Suh et al. (2021)
Baringo mice	deafness	AAV	AID-CBEmax	*Tmc1*	inner ears	Yeh et al. (2020)
Dmd knockout mice	Duchenne muscular dystrophy	AAV	ABE7.10	*Dmd*	skeletal muscles	Ryu et al. (2018)
mdx4cv mice	Duchenne muscular dystrophy	AAV	AAV-iNG	*Dmd*	heart, gastrocnemius, diaphragm and muscles	Xu et al. (2021)
∆E51 mice	Duchenne muscular dystrophy	AAV	ABEmax	*Dmd*	muscles	Chemello et al. (2021)
			PE			
SC-SMA^∆7^ mice	spinal muscular atrophy	Plasmid	miniABEmax	*Smn2*	lateral ventricles	Lin et al. (2020)
β-YAC/CD46 mice	β-hemoglobinopathies	Adenovirus	ABE	*HBG1 and HBG2 promoter*	bone marrow cells	Li et al. (2021)
Npc1tm (^I1061T^) mice	Niemann–Pick disease type C	AAV	CBE3.9max	*Npc1*	cortex cerebellum	Levy et al. (2020)
			ABEmax			
G93A-SOD1 mice	Amyotrophic lateral sclerosis	AAV	BE4	*Sod1*	spinal cord	Lim et al. (2020)
Fah−/− mice	Hereditary tyrosinaemia type I	LPN	ABE6.3 RA6.3	*Fah*	liver	Song et al. (2020)
Fah−/− mice	Hereditary tyrosinemia type 1	AAV	BE3	*Hpd*	liver	Rossidis et al. (2018)
NSG-PiZ mice	alpha-1 antitrypsin deficiency	LPN	BE4	*Serpina1*	liver	Packer et al. (2022)
(Pah)enu2 mice	phenylketonuria	AAV	BE3	*Pah*	liver	Villiger et al. (2018)
B6.BTBR-Pahenu2	phenylketonuria	AAV	BE-PLUS	*Pah*	liver	Zhou et al. (2022)
HGPS mice	Hutchinson–Gilford progeria syndrome	AAV	ABE7.10max-VRQR	*LMNA*	aorta	Koblan et al. (2021)
					bone	
					muscle	
					liver	
Idua-^W392X^ mice	Hurler syndrome	AAV	ABEmax	*Idua*	liver	Bose et al. (2021)

**TABLE 2:** *In vitro/ex vivo* DNA editing for therapy of monogenic diseases.

Cell line/Primary cells	Model	Delivery system	Editing systems	Target gene	References
LCL^HFEC282Y^	haemochromatosis	Plasmid	ABE7.10	*HFE*	Gaudelli et al. (2017)
HEK293T^HBG1/HBG2 (−113mut,−175mut and −198mut)^	β-hemoglobinopathies	Plasmid	ABEmax	*HBG1/2*	Koblan et al. (2018)
HEK293T^HBG1/HBG2 (−175mut and −198mut)^	β-hemoglobinopathies	Plasmid	ABE8e	*HBG1/2*	Richter et al. (2020)
CD34^+^ cells from donors with Sickle-cell disease	β-hemoglobinopathies	Plasmid	ABE8 variants	*HBG1/2*	Gaudelli et al. (2020)
Fibroblast cells from β-thalassemia patients	β-hemoglobinopathies	Plasmid	BE3	*HBB*	Liang et al. (2017)
β-thalassemia patient-derived erythroid precursor cells	β-hemoglobinopathies	Plasmid	eA3A-BE3	*HBB*	Gehrke et al. (2018)
CD34^+^ cells from a β-thalassemia patient	β-hemoglobinopathies	RNP	hA3A-BE3	*HBB*	Liren Wang et al. (2020)
HUDEP-2 cells	β-hemoglobinopathies	Plasmid	A&C-BEmax	*HBG1/2* promoter	Zhang et al. (2020)
CD34^+^ hematopoietic stem and progenitor cells derived from β-thalassemia patient	β-hemoglobinopathies	RNP	A3A (N57Q)-BE3	*HBG1/2* and *HBB* promoter	Zeng et al. (2020)
CD34^+^ cells from donors with Sickle-cell disease	β-hemoglobinopathies	RNP	ABE8e-NRCH	*HBB*	Newby et al. (2021)
HEK293T ^HBB (G6V)^	β-hemoglobinopathies	Plasmid	ABE8e-NRCH	*HBB*	Miller et al. (2020)
HEK293T^HBG1/HBG2 (−198T/C)^	β-hemoglobinopathies	Plasmid	ABE7.10	*HBG1/2*	Gaudelli et al. (2017)
CuFi-3 (CFTR R553X) primary cells derived from Cystic fibrosis-affected individuals	Cystic fibrosis	RNP	ABE7.10-NG	*CFTR*	Krishnamurthy et al. (2021)
FBN1^T7498C^ cells	Marfan Syndrome	Plasmid	BE3	*FBN1*	Zeng et al. (2018)
Human embryos FBN1^T7498C^ (2d)		Microinjected mRNA of BE3 and sgRNA into zygotes			
chemically derived hepatic progenitors (CdHs)	Hereditary tyrosinemia type 1	Plasmid	ABEmax	*Fuh*	Kim et al. (2021)
			PE3		
Mouse astrocytes (APOE4)	Alzheimer’s disease	Plasmid	BE3	*APOE*	Komor et al. (2016)
HEK293T and SH-SY5Y cells	Alzheimer’s disease	Plasmid	Target-AID	*APP*	Guyon et al. (2021)
HEK293T^HBB(E6V)^	Sickle cell disease	Plasmid	PE3	*HBB*	Anzalone et al. (2019)
HEK293T^HEXA (1278+TATC)^	Tay-Sachs syndrome	Plasmid	PE3	*HEXA*	Anzalone et al. (2019)
HEK293T^PRNP(G127V)^	Prion disease	Plasmid	PE3	*PRNP*	Anzalone et al. (2019)
Patient-derived fibroblasts harboring the MPDU1^L119P^	congenital disorder of glycosylation type 1f	Plasmid	BE4max	*MPDU1*	Koblan et al. (2018)
N2a neuroblastoma cells	Chronic pain	Plasmid	BE4max	*SCN9a*	Koblan et al. (2018)
Derived from children with progeria	Hutchinson–Gilford progeria syndrome	lentivirus	ABE7.10max-VRQR	*LMNA*	Koblan et al. (2021)

### In vivo

#### Eye diseases

DNA-editing techniques have been successfully applied *in vivo* to correct the *Rpe65* gene mutation that is the cause of Leber congenital amaurosis (Jo et al., 2021; Suh et al., 2021). *Rpe65* codes an enzyme that is essential for the conversion of vitamin A from all-*trans*-retinol to 11-*cis*-retinal: the chromophore of the visual pigments. Consequently, a loss of the functional RPE65 enzyme leads to severe visual impairment from birth or in the first several months of life but does not affect other tissues and organs (Chao et al., 1993). In two refs. (Jo et al., 2021; Suh et al., 2021), in murine strain *rd12* (carrying a nonsense mutation in exon 3: c.130C > T; p.R44X), which manifests the first signs of retinal degeneration at ∼3 weeks of age (Pang et al., 2005), investigators were able to rescue retinal and visual function. Suh and others (Suh et al., 2021) have tested the ABEmax system, which they have delivered into retinal cells by means of a lentivirus and achieved the following: RPE65 expression is restored in 32% of retinal cells, the total amount of 11-*cis*-retinal is 30% of the level in wild-type mice, and there is a 34% reduction in the concentration of all-*trans-*retinyl esters. In another study (Jo et al., 2021), investigators used the NG-ABEmax system as well as a dual AAV with trans-splicing intein as a vector for delivery to retinal cells; however, the efficiency of DNA editing was lower (13%) than that in ref. (Suh et al., 2021).

### Hearing loss–related diseases

Neonatal injection of dual AID-CBEmax AAVs into the inner ears of deaf Baringo mice carrying point mutation A545G in the *Tmc1* gene, coding for transmembrane channel-like one protein, has helped to restore inner hair cell sensory transduction and hair cell morphology and transiently rescued low-frequency hearing 4 weeks after the injection (Yeh et al., 2020).

### Neuromuscular disorders

Adenine base editing has been successfully used to correct the *DMD* gene (dystrophin) mutations that are associated with Duchenne muscular dystrophy (Ryu et al., 2018; Xu et al., 2021). Loss of dystrophin leads to progressive muscle weakness and wasting, which eventually leads to respiratory disturbances, cardiomyopathy, and death before the age of 30 (Mercuri and Muntoni, 2013). The first DNA editing by means of the ABE7.10 system was performed in 2018 (Ryu et al., 2018): researchers performed intramuscular administration of two *trans*-splicing AAV vectors into the *tibialis anterior* muscle in *Dmd* knockout mice (carrying a nonsense mutation in exon 20) and evaluated therapy efficacy at 8 weeks postinjection. Postmortem histological analysis of the *tibialis anterior* showed that dystrophin expression was restored in 17% of myofibers, and deep sequencing analysis revealed that the efficiency of editing was ∼3.3%. These results are encouraging because ∼4% of normal dystrophin expression is sufficient to improve muscle function (Putten et al., 2013). In 2021 single systemic administration of the iABE-NGA system delivered by means of two AAVs (via tail vein injection) was performed to restore dystrophin in *mdx* ^*4cv*^ mice, which carry a premature stop codon in exon 53 (Xu et al., 2021). A distinctive feature of the study is that it covered two time points (∼5 weeks and ∼9 months postadministration), which helped to evaluate the long-term impact of systemic ABE editing therapy. The results indicate that the effects of single administration of the systemic ABE editing therapy even strengthen with time in some tissues: there was 45.9% restoration of dystrophin levels in the heart at 5 weeks compared to wild-type mice and 95.9% at 9 months, ∼10% restoration in the gastrocnemius at 5 weeks and ∼5% at 9 months, as well as ∼4% restoration in the diaphragm at 5 weeks and ∼8% at 9 months. Similar results of systemic DNA editing in various organs have been obtained after a single retro-orbital injection of the CBE3.9max system, which is designed to edit a silent mutation in the murine *Dnmt1* locus (editing efficiency up to 59% in the brain, up to 38% in the liver and retina, up to 9% in skeletal muscle, and up to 20% in the heart) (Levy et al., 2020). Finally, PE editors have also been used successfully for the treatment of muscular dystrophy (Chemello et al., 2021).

Furthermore, base editors have been employed to treat spinal muscular atrophy (Lin et al., 2020). The latter is a progressive motor neuron disease (caused by a mutation in the *SMN1* gene) with onset during infancy and causes motor impairments and death in the first years of life. Neonatal injection of the miniABEmax system into lateral ventricles of SMNΔ7 SMA mice yielded an editing efficiency of 3–5% on postnatal day 7.

### Blood disorders

Base-editing tools have been successfully applied to treat β-thalassemia *in vitro*, *ex vivo*, and *in vivo* (Gaudelli et al., 2017, 2020; Liang et al., 2017; Gehrke et al., 2018; Koblan et al., 2018; Wang L. et al., 2020; Miller et al., 2020; Richter et al., 2020; Zeng et al., 2020; Zhang et al., 2020; Antoniou et al., 2021; Li et al., 2021; Newby et al., 2021). β-Thalassemia develops due to deficient production of β-globin and is characterized by microcytic hypochromic anemia and abnormal results on a peripheral-blood smear. Reactivation of γ-globin expression is associated with lowered morbidity and mortality and significantly relieved disease symptoms. Therefore, therapeutic strategies against β-thalassemia are based both on the correction of a mutation in the *HBB* gene and on the introduction of mutations that disrupt binding sites of repressor proteins or create gain-of-function binding sites for activators, thereby derepressing γ-globin expression. For example, in an *in vitro* experiment on HUDEP-2 (ΔGγ) cells (Zhang et al., 2020) using the A&C-BEmax system, investigators disrupted the BCL11A binding site (strong transcription repression element) in the promoter of γ-globin genes (*HBG1* and *HBG2*) and generated a GATA1-binding site (active as enhancer) *de novo* in the promoter. Zhang and others have been able to achieve over 40% editing efficiency and nearly sixfold enhancement of γ-globin mRNA expression as compared to its expression in HUDEP-2 cells. *Ex vivo* ribonucleoprotein electroporation of the A3A^(N57Q)^-BE3 system into human-peripheral-blood-mobilized CD34^+^ hematopoietic stem and progenitor cells also successfully disrupts a GATA1-binding motif and reduces BCL11A expression. In addition, those authors edited the HBB −28A>G promoter mutation. Due to this multiplex approach, substantial efficiency of DNA editing and upregulation of β- and γ-globins were achieved (Zeng et al., 2020). An *in vivo* experiment (Li et al., 2021) has been performed on β-YAC^+/−^/CD46^+/+^ mice, which were obtained by crossing mice carrying a yeast artificial chromosome (β-YAC) bearing the wild-type 248-kbp human β-globin locus with homozygous transgenic mice expressing human CD46. This approach is based on transduction of peripheral CD34^+^ hematopoietic stem/progenitor cells (for intravenous injection) with an adenovirus containing ABE vectors. CD34^+^ hematopoietic stem/progenitor cells are mobilized beforehand in β-YAC^+/−^/CD46^+/+^ mice by subcutaneous injections. The transduced cells return to bone marrow, where they persist long-term. Base editing in hematopoietic stem/progenitor cells by means of an ABE vector led to efficient γ-globin induction, which persisted for 16 weeks after the introduction of the genetic construct. Another successful example of directed base-editing is the *in vivo* correction of mutated GTG (Val) codon encoding amino acid 6 in β-globin gene (*HBB* ^*S*^), which leads to sickle cell disease (SCD) (Newby et al., 2021). Authors combine the engineered Cas9-NRCH nickase, that recognizes a CACC protospacer-adjacent motif, with deoxyadenosine deaminase TadA-8e to generate ABE8e-NRCH. This base editor was transfected into human CD34^+^ HSPCs from SCD donors or mice *HBB* ^*S/S*^ HSPCs via electroporation of ABE8e-NRCH and sgRNA in RNA or RNP forms. The editing resulted in formation of Makassar β-globin (*HBB* ^*G*^), a non-pathogenic variant with alanine in sixth position. Edited human cells were transplanted in immunodeficient NBSGW mice that support multilineage engraftment of human hematopoietic cells. This resulted in a decrease of β^S^ from 96 ± 0.28% of total β-like globin protein in unedited erythroblasts to 40 ± 2.3% in edited erythroblasts. The amount of β^G^ in edited cells reached 58 ± 2.8%. The electroporation of ABE8e-NRCH RNP into mice *HBB* ^*S/S*^ HSPCs followed by transplantation into irradiated adult recipient mice led to expression of βG that made up 75–82% of total β-like globin protein. Moreover, transplantation of base-edited *HBB* ^*S/S*^ HSPCs restored all tested blood parameters to levels similar to those of healthy control mice (Newby et al., 2021). Of note, such *in vivo* rodent experiments on human cell lines allow investigators to adapt editing tools for future clinical trials.

### Neurodegenerative disorders

In this field, the first step toward the treatment of neurodegenerative diseases was recently made in *Npc1* ^I1061T^ mice, which are a model of Niemann–Pick disease, also known as neurodegenerative ataxia (Levy et al., 2020). A nonsense mutation in the intracellular cholesterol transporter (*Npc1*) gene leads to impaired cholesterol trafficking and accumulation of cholesterol inside cells. Niemann–Pick disease features ataxia, motor impairment, progressive intellectual disability, and dementia (Praggastis et al., 2015). A single retro-orbital injection of the CBE3.9max system into *Npc1* ^I1061T^ mice prolonged the survival of Purkinje neurons and caused a 10% increase in the lifespan of the mice as compared with untreated mice.

Base-editing tools have also been tested in the treatment of amyotrophic lateral sclerosis in *SOD1* ^*(G93A)*^ mice, which are characterized by an especially aggressive course of the neurodegenerative disease and have an average lifespan of ∼120 days (Lim et al., 2020). Amyotrophic lateral sclerosis is an autosomal dominant disease, and some cases are associated with a defective protein, superoxide dismutase 1 (SOD1), whose accumulation leads to a loss of motor neurons in the spinal cord and brain (Rosen, 1993). Intrathecal injection of dual AAV particles encoding a split-intein CBE system improved motor functions, reduced mutant-SOD1 reactive inclusions in the spinal cord, and increased the animals’ lifespan by ∼11%. Nevertheless, overall editing efficiency was ∼1.2%, and only ∼6.5% of spinal-cord cells were successfully transduced by both AAV vectors, suggesting that there is some room for improvement of the therapy efficacy (Lim et al., 2020).

### Metabolic disorders

Various DNA-editing approaches have been implemented for the treatment of type I hereditary tyrosinemia, which is attributed to loss of function of fumarylacetoacetate hydrolase (FAH) (Rossidis et al., 2018; Song et al., 2020; Kim et al., 2021). FAH deficiency impairs tyrosine catabolism, induces accumulation of toxic metabolic intermediates in the liver, and has a cytotoxic effect on hepatocytes (Grompe, 2001). *Fah* ^*mut/mut*^ mice have a mutation in exon eight of the *Fah* gene, whereas treatment of adult mice with either the ABE6.3 or RA6.3 system restores the expression of functional FAH in ∼1% and ∼4% of hepatocytes, respectively (Song et al., 2020), and as a consequence, *Fah* ^*mut/mut*^ mice do not experience the characteristic weight loss after discontinuation of administration of a tyrosine-catabolic pathway inhibitor: 2-(2-nitro-4-trifluoromethylbenzoyl)-1,3-cyclohexanedione. Those authors applied two nonviral strategies for systemic delivery of the editing systems via tail vein injection: hydrodynamics-based transfection of plasmid DNA and lipid nanoparticle–mediated delivery of mRNAs, and the efficiency of the former was almost an order of magnitude higher. In ref. (Rossidis et al., 2018), scientists attempted to edit DNA during the embryonic period. Unlike previous studies aimed at restoring the function of a protein, here the focus was on introducing a nonsense mutation into 4-hydroxyphenylpyruvate dioxygenase (*Hpd*). Inactivation of HPD in *Fah* ^*mut/mut*^ mice prevents the accumulation of toxic metabolites of tyrosine in the liver. An adenoviral vector was used to deliver the BE3 editing system, which was injected into the vitelline vein on fetal day 16. The efficiency of base editing in the liver was found to gradually increase: 14% on postnatal day 1, 37% on postnatal day 30, and 40% on postnatal day 90. In this way, in these mice, aspartate aminotransferase (AST), alanine aminotransferase (ALT), and serum bilirubin levels and the number of hepatocytes were restored. Therefore, embryonic DNA editing holds promise as a therapeutic modality for complex genetic disorders identified during prenatal screening.

*In vivo* models based on NSG-PiZ mice have been utilized to successfully correct alpha-1 antitrypsin deficiency, which is characterized by a lung disease and/or liver disease (Packer et al., 2022). Mutations in the SERPINA1 gene induces misfolding of the protein product and accumulation of toxic aggregates within hepatocytes, along with insufficient inhibition of neutrophil elastase in lungs (Fregonese and Stolk, 2008). Tail vein injection of the BE4 system as lipid nanoparticle–based formulation of RNA into adolescent NSG-PiZ mice has exerted pronounced effects already at 1 week after treatment (histological changes in the liver and biochemical alterations in blood serum), and these characteristics only improved at 12 and 32 weeks after this therapy (Packer et al., 2022).

Another example of the use of base editing for the correction of mutations that lead to metabolic disorders is a treatment of phenylketonuria in (Pah)^enu2^ mice (Villiger et al., 2018; Zhou et al., 2022). Phenylketonuria is characterized by phenylalanine hydroxylase deficiency and impaired metabolism of L-Phe, resulting in systemic hyperphenylalaninemia. Without an appropriate dietary therapy, this condition causes damage to the central nervous system and induces severe intellectual disability (Blau et al., 2010). A three-stage study included application of the BE3 system to cultured cells, to liver organoids, and to (Pah)^enu2^ mice. In the *in vivo* experiment, L-Phe blood levels and mRNA correction rates were time- and dose-dependent, peaking at 26 weeks after injection (conversion up to 63%). It must be pointed out that only a high concentration of the AAV (5 × 10^11^ *v*g vs. 1 × 10^11^ *v*g) was able to return the blood level of L-Phe to the physiological range (below 120 μmol/L). In another study (Zhou et al., 2022), researchers performed intravenous injection of AAV vectors carrying the BE3-PLUS editing system on the second postnatal day and achieved a sustained dose-dependent reduction in blood L-Phe levels up to 24 weeks of age.

Of note, base editors are also employed to inactivate genes that have unwanted functions. A vivid example is the *PCSK9* gene: a loss-of-function mutation in this gene results in reductions of the enzyme and low-density lipoprotein cholesterol levels; the latter change has a protective effect, i.e., reduces the risk of atherosclerotic cardiovascular disease (Rao et al., 2018). Base editors that are delivered *in vivo* using different delivery systems (a lipid nanoparticle–based adenoviral vector or AAV) can efficiently knock down *PCSK9* in the liver after a single infusion, with concomitant stable reductions in blood levels of PCSK9 and low-density lipoprotein cholesterol in mice and monkeys (Carreras et al., 2019; Musunuru et al., 2021; Rothgangl et al., 2021).

### Other genetic disorders

Comprehensive research on DNA-editing tools has been conducted to treat Hutchinson–Gilford progeria syndrome (HGPS) in mice (Koblan et al., 2021). Hutchinson–Gilford progeria is an autosomal dominant disease featuring rapid aging, cardiovascular disease, and early death. The illness is caused by a point mutation in the lamin A (*LMNA*) gene; this mutation leads to a mis-splicing event and to the formation of a truncated nonfunctional progerin protein (De Sandre-Giovannoli et al., 2003). Investigators conducted both an *in vitro* experiment on fibroblast lines derived from patients with HGPS and an experiment on a mouse model transgenic for human *LMNA* (HGPS mice); these mice exhibit symptoms of cardiovascular complications and have a life expectancy of ∼215 days. The *in vitro* experiment involving lentiviral delivery of the ABE7.10max-VRQR system yielded up to 90% genomic correction of the *LMNA* mutation and a ≤90% reduction in progerin levels both at 10 and 20 days after administration. The experiment on mice was carried out via systemic retro-orbital injections of the ABE7.10max-VRQR system. In the *in vivo* experiment, in contrast to the *in vitro* assay, the delivery was implemented using two AAV9 vectors with trans-splicing inteins, which have broad tissue tropism. The researchers tested several time points of treatment administration—a single injection on the third or 14th postnatal day—as well as long-term effects (analysis of the results at ages 6 weeks and 6 months). The results indicated that regardless of age (6 weeks or 6 months), the efficiency of DNA editing in the target organs (the heart, liver, aorta, and bone) persists and reaches 10–60% depending on the organ, whereas the amount of progerin decreases by ≤ 90%. Histological analysis showed that the treatment with the ABE system significantly alleviates morphological manifestations of the disease: a modestly reversed loss of the hypodermal fat layer and an increase in the number of vascular smooth muscle cells in the aorta by more than threefold. Besides, the treated HGPS mice had a lifespan 1.8–2.4 times that of untreated HGPS mice. Moreover, the results of the ABE therapy on the 14th postnatal day were significantly better in various parameters as compared with the therapy on postnatal day 3. On the other hand, as the authors themselves stated, such effects may be explained by the ∼10-fold higher dose of AAVs (the dose was calculated based on body weight). Nevertheless, questions about the optimal age for the therapy of various genetic diseases and about the optimal dose of the therapeutic vector remain open.

A nonsense mutation in the *IDUA* gene leads to the absence of the corresponding enzyme (αl-iduronidase) and a buildup of large sugar polymers (glycosaminoglycans) in lysosomes, thus inducing one form of type 1 mucopolysaccharidosis (Hurler syndrome). In ref. (Bose et al., 2021), *in utero* and postnatal base editing by ABE improved cardiac function and survival of Idua-W392X mice. The adult mice demonstrated normalization of biochemical, histological, and neurobehavioral parameters, with a more pronounced recovery in the mice treated embryonically.

### In vitro

DNA-editing technologies for the treatment of facioscapulohumeral muscular dystrophy, cystic fibrosis, prion diseases, sickle cell disease, Alzheimer’s disease, and Tay-Sachs and Marfan syndromes have so far been tested only on *in vitro* models (cell culture) (Anzalone et al., 2019; Guyon et al., 2021; Krishnamurthy et al., 2021; Šikrová et al., 2021). The first study on a cytosine base editor (BE3) was published in 2016 (Komor et al., 2016). The editing was targeted to point mutations in the *APOE* gene, whose sequence alterations significantly increase the risk of Alzheimer’s disease. Nucleofection of the BE3 system into immortalized mouse astrocytes—in which the endogenous *Apoe* gene was replaced by human *APOE4*—resulted in 58–75% efficiency of DNA editing. Editing of another point mutation that is also associated with the risk of Alzheimer’s disease—a substitution in the amyloid precursor protein (*APP*) gene—by means of the BE3 system reduced the amounts of Aβ_40_ and Aβ_42_ peptides *in vitro* by more than 20% (Guyon et al., 2021).

Another research project (Šikrová et al., 2021) is based on immortalized myoblasts derived from individuals susceptible to facioscapulohumeral muscular dystrophy (two subtypes: FSHD1 and FSHD2). To suppress unwanted expression of DUX4, an approach was used involving the introduction of a mutation into the functional polyadenylation signal (ATTAAA) in an exon with the help of the ABEmax system. The findings revealed a significant decrease in *DUX4* mRNA levels (10–1,000-fold downregulation).

A mutation in the cystic fibrosis transmembrane conductance regulator (*CFTR*) gene causes an inherited disorder that involves severe damage to the lungs, digestive system, and other organs. One study (Krishnamurthy et al., 2021) — performed on both the CuFi-3^(CFTR R553X)^ cell line and primary human airway epithelial cells with specific CFTR mutations—has revealed successful DNA editing (up to 80% efficiency) and restoration of CFTR anion channel function by means of ABE7.10-NG systems.

An example of successful DNA editing during the embryonic period involves the heterozygous mutation T7498C in the fibrillin gene (*FBN1*) in human embryos (Zeng et al., 2018). Pathogenic *FBN1* mutations cause Marfan syndrome, which is an autosomal dominant disease that affects the skeletal, ocular, and cardiovascular systems. Researchers microinjected mRNA of the BE3 system into zygotes and after 2 days evaluated the effectiveness of the therapy. Additionally, an experiment was conducted on modified cell line HEK293T (FBN1T^7498C^). In both cases, high efficiency of targeted editing was achieved (40–90%).

Finally, the use of the PE3 system in cell lines with various mutations—HEK293T^HBB(E6V)^ (a model of sickle cell disease), HEK293T^HEXA(1278+TATC)^ (a model of Tay-Sachs syndrome), and HEK293T^PRNP(G127V)^ (a model of a prion disease)—has yielded high editing efficiency (31–53%) and low numbers of indels (<5%) (Anzalone et al., 2019). Collectively, these data imply that the PE3 system can either introduce or correct transversion, insertion, or deletion mutations.

### Translational potential of site-directed DNA-editing systems for gene therapy of monogenic diseases

There are few successfully implemented clinical trials of the CRISPR-Cas9 system, and site-directed editing systems based on dCas9 or nCas9 for the treatment of rare monogenic disease have so far been tested *in vivo* only on rodents. The absence of clinical trials of these systems is probably due to the fact that they were discovered relatively recently. The first successful case of CRISPR-Cas9 application *in vivo* involves the treatment of patients with hereditary transthyretin amyloidosis, which is characterized by accumulation of amyloid fibrils in tissues. Intravenous administration of CRISPR-Cas9 prevented the synthesis of the defective protein through frameshift mutations (trial registration # NCT04601051) (Gillmore et al., 2021). Furthermore, the CRISPR-Cas9 system is currently being tested in a clinical trial (NCT03872479) on 18 patients with type 10 Leber congenital amaurosis and is aimed at removing a point mutation in the *CEP290* gene. CRISPR-Cas9 has found broader applications in *ex vivo* clinical studies (Arnold, 2021; He et al., 2021). In particular, CRISPR-Cas9–edited HSPCs with inactivated BCL11A (a transcription factor responsible for the repression of fetal hemoglobin expression) (clinical trials NCT03745287 and NCT00842634) had significantly ameliorated the manifestations of sickle cell disease and transfusion-dependent β-thalassemia, and the effects were stable for more than a year (Frangoul et al., 2021). These examples indicate that a Cas9-based tool, similar to dCas9-based and nCas9-based systems, can be utilized to restore a normal gene sequence, to create a landing site for a transcription factor, and to inactivate a gene. Of note, clinical-trial data so far are still preliminary and derive from an analysis of a very small number of patients with limited follow-up. The issue of long-term consequences of possible off-target effects and indels remains unresolved too; these are natural outcomes of DSB repair (Lin et al., 2014; Meyenberg et al., 2021). It has been shown, that base editing of human HSPCs avoided p53 activation (Newby et al., 2021). The activation of p53 leads to different cellular outcomes such as cell cycle arrest and apoptosis; the former facilitates DNA repair and promotes cell survival (Zhang et al., 2011). Overall, replacing CRISPR-Cas9 editors with nCas9-based or dCas9-based DNA-editing systems, which do not produce a DSB, looks promising. An analysis of clinical trials in diseases against which CRISPR-Cas9 has been used to date suggests that such a replacement is possible from a functional point of view, but the limitations of dCas9-based and nCas9-based systems should be addressed.

The main requirement for the use of base editors is targetability, whose disruption gives rise to off-target effects (Komor et al., 2016; Gaudelli et al., 2017). The number of off-target effects depends on PAM specificity, single guide RNA design, deaminase DNA- or RNA-binding capacity, and Cas variants (Zuo et al., 2019; Huang et al., 2021). It is worth mentioning that the deaminases that form the basis of DNA base editors have an activity toward RNA bases, and the APOBEC family of deaminases and DNA editors based on them are especially nonspecific (Levy et al., 2020). TadA deaminase, which is a part of ABE systems, is more specific, and therefore ABE systems lead to significantly fewer *de novo* single-nucleotide variants (Levy et al., 2020). On the other hand, ABE systems also tend to convert cytosine to guanine or thymine, and these substitutions occur independently of adenosine conversions (Kim et al., 2019). This ABE-mediated cytosine conversion is single-guide-RNA–dependent and may be minimized via improvement of the guide RNA by chemical modifications (Kim et al., 2019).

In addition, the number of off-target effects strongly depends on the method of delivery of the genetically engineered construct (Lin et al., 2022). The main means of delivery of CRISPR-Cas9–based editors are DNA constructs (plasmids or genetic cassettes of a viral vector) or ribonucleoprotein complexes, which differ in editing efficiency and lifetime. The advantage of base-editing systems involving CRISPR-Cas9 over gene-adding strategies and RNA editors is a permanent effect. Accordingly, treatment with base editors in the form of short-lived ribonucleoproteins can produce a stable therapeutic outcome that can last for life.

## Conclusion

Findings from *in vivo* experiments suggest that even single systemic administration of a base editor can have long-term numerous effects on many tissues and organs, thereby significantly increasing life expectancy, thus making this strategy feasible even in the treatment of the most complicated genetic diseases. Rapid developments in base-editing systems are intended to reduce off-target effects and raise editing efficiency. One of the most promising approaches in this context is the improvement of bioinformatic approaches [for instance, based on a deep learning algorithm that is capable of predicting base-editing outcomes (Marquart et al., 2021)] that would help to select optimal editing tools on the basis of individual genetic characteristics of a patient. We believe that the optimization of base-editing tools and the design of new bioinformatic approaches will enable the testing of these tools in clinical trials in the next 5 years.
